# Impact of COVID-19 Infection on Liver Transplant Recipients: Does It Make Any Difference?

**DOI:** 10.7759/cureus.22687

**Published:** 2022-02-28

**Authors:** Daniela Punga, Sebastian Isac, Cristian Paraipan, Mihail Cotorogea, Andreea Stefan, Cristian Cobilinschi, Ileana Adela Vacaroiu, Raluca Tulin, Dorin Ionescu, Gabriela Droc

**Affiliations:** 1 Department of Anesthesiology and Intensive Care I, Fundeni Clinical Institute, Bucharest, ROU; 2 Department of Anesthesiology and Intensive Care, Clinical Emergency Hospital, Bucharest, ROU; 3 Department of Nephrology, Faculty of Medicine, Carol Davila University of Medicine and Pharmacy, Bucharest, ROU; 4 Department of Anatomy and Embryology, Carol Davila University of Medicine and Pharmacy, Bucharest, ROU; 5 Department of Endocrinology, Emergency Hospital Prof. Dr. Agrippa Ionescu, Bucharest, ROU; 6 Department of Internal Medicine and Nephrology, Faculty of Medicine, Carol Davila University of Medicine and Pharmacy, Bucharest, ROU

**Keywords:** severity, outcome, liver transplant, immunosuppression, covid-19

## Abstract

The first case of coronavirus disease 2019 (COVID-19) was diagnosed in December 2019 in Wuhan, China. Since then, this novel infectious disease, caused by the severe acute respiratory syndrome coronavirus type 2 (SARS-CoV-2), has grown into a pandemic with over 330 million infected individuals worldwide, many of them with innate or acquired immunosuppression.

Liver transplantation (LT) is offered as a curative therapy for end-stage liver disease as well as for acute liver failure cases. Advances in immunosuppressive therapy decreased the rates of acute and chronic graft rejection, significantly improving the quality of life.

Liver transplant recipients are considered at particularly high risk for developing critical COVID-19 infection because of their chronic immunosuppressed state. Available data are heterogeneous, and the mortality rate is variably reported in the literature. There is controversy regarding whether their immunosuppressive status is a risk or a protective factor for developing severe respiratory disease. Moreover, the mechanism of action is still unclear.

We report the clinical outcome of three liver transplant recipients who had COVID-19 pneumonia at different moments following liver transplantation. All patients received a standard immunosuppression regimen and specific antiviral therapy, requiring no invasive mechanical ventilation. They were discharged from the hospital with no long-term COVID-19 complications.

## Introduction

Since the beginning of the severe acute respiratory syndrome coronavirus type 2 (SARS-CoV-2) pandemic, millions of people became victims worldwide [1-3]. Considered to be at risk, the immunosuppressed patients represent a priority for all medical facilities confronted with this pandemic. These patients are more susceptible to severe acute respiratory syndrome due to SARS-CoV-2 [4]. Moreover, such patients are an ideal host for promoting the mutagenic potential of this virus. Thus, highly mutated variants are indicative of a form of rapid, multistage evolutionary jump, which could preferentially occur in the milieu of incomplete immune control [4]. 

The presence of a large number of mutations is also a hallmark of the variants of concern, including B.1.1.7 (alpha), B.1.351 (beta), P.1 (gamma), and B.1.617.2 (delta), which suggests that the viral evolution in immunocompromised patients may be an important factor in the emergence of such variants [4]. Since many people globally are living with innate or acquired immunosuppression, the association between immunosuppression and the generation of highly transmissible or more pathogenic SARS-CoV-2 variants requires further delineation and mitigation strategies.

Moreover, liver transplant recipients raise obvious concerns worldwide as a consequence of the interaction between their acquired immunosuppression and SARS-COV-2 infection, which makes them particularly vulnerable. They are mostly referred to specialized liver surgery centers [5,6]. Conversely, these medical centers have limited capacity to manage lung infections specific to this pandemic era. 

Standard therapy in liver transplantation generally involves a mixture of corticosteroids, calcineurin inhibitors (CNI) (cyclosporine or tacrolimus), and antiproliferative agents (mycophenolate mofetil {MMF}), in accordance with local guidelines [7]. The intensity of the immunosuppression required in liver transplantation is, however, different from other pathologies, which need primary prophylaxis against graft rejection [8]. The immunosuppression therapy is also adjusted taking the time elapsed since the surgical procedure into consideration.

A paucity of data is available regarding coronavirus disease 2019 (COVID-19) pneumonia in this particular subpopulation, and only few articles are focusing on the severity of COVID-19 pneumonia correlated with the immune status of a liver-transplanted patient with regard to the time elapsed since the initial surgery [9]. Thus, further efforts should be made in order to establish if the patient outcome secondary to COVID-19 pneumonia could be influenced by this additional immunological condition [10]. Thus, our study aimed to address this issue by describing a case series of three liver-transplanted patients who acquired COVID-19 at different time points from the liver transplantation and their outcomes.

## Case presentation

Case 1

A 64-year-old male patient underwent an orthotopic liver transplant for decompensated liver cirrhosis secondary to hepatitis C virus and grafted hepatocellular carcinoma, in accordance with the Milan criteria for liver transplantation [11]. His medical records revealed a major right bundle branch block, mild mitral and tricuspid regurgitations, first-degree esophageal varices, a renal cyst, and erythematous gastritis.

**Figure 1:**
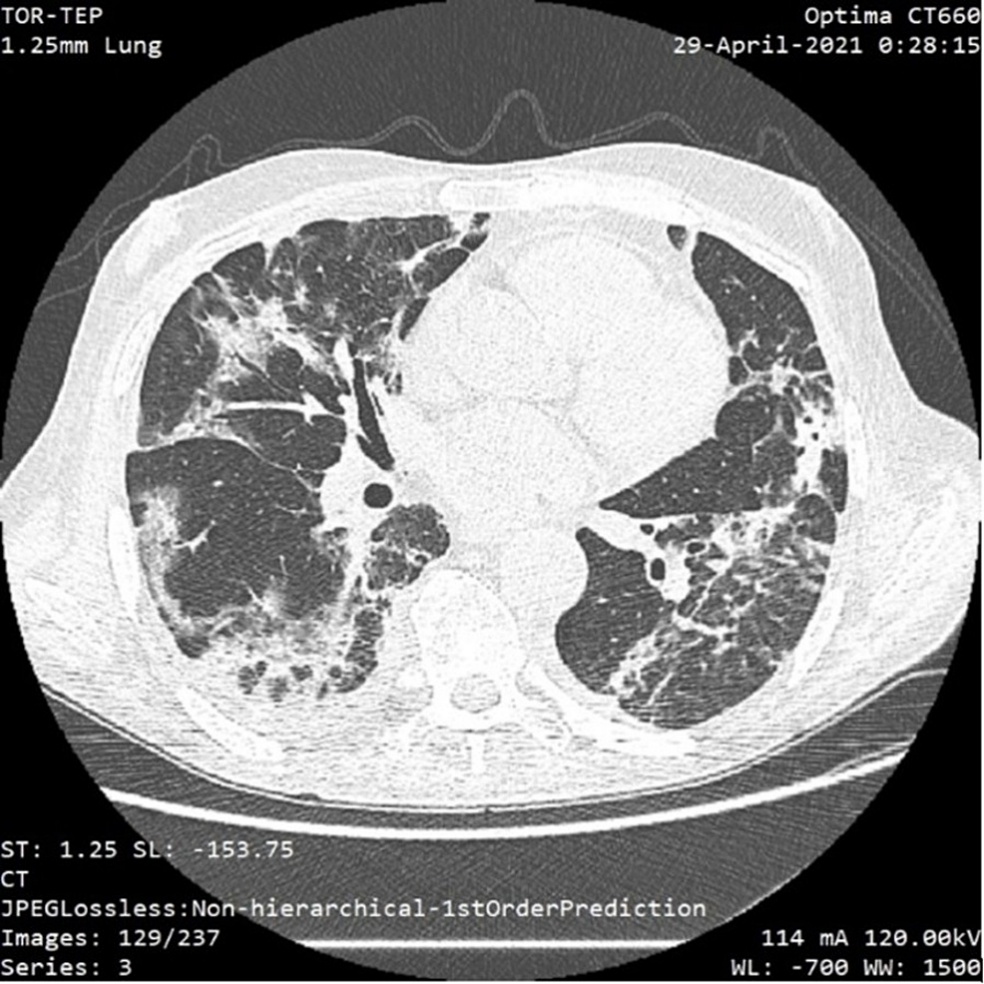
CT scan showing minimal bilateral pleural effusions and typical glass opacities and fibrosis in approximately 35% of the lung parenchyma

The patient received 20 mg basiliximab and 500 mg methylprednisolone intraoperatively. Further, the patient was administered a combination of tacrolimus and mycophenolate mofetil, in accordance with the blood count analysis and liver function tests. A second dose of basiliximab was administered on the fourth postoperative day. The patient was discharged after a favorable postoperative evolution, having been prescribed oral immunosuppression therapy with tacrolimus. Four months after the liver transplantation, the patient was transferred to our clinic with acute hypoxemic respiratory failure secondary to COVID-19 infection. The CT findings are illustrated in Figure 1 showing 35% lung involvement.

Despite the moderate-to-severe form of COVID-19 pneumonia, the patient didn’t require management in an intensive care unit (ICU). The patient received supplemental oxygen over a non-rebreather oxygen mask with a maximum flow of 8 l/min. The patient received 10 days of specific antiviral therapy, comprising remdesivir and corticosteroids, while the chronic immunosuppression with oral tacrolimus was continued, with a dosing regimen adjusted in accordance with the plasma concentration. The patient was discharged from the hospital 21 days after the positive SARS-COV-2 test, with no other respiratory features.

Case 2

A 56-year-old female patient underwent a living donor transplant for decompensated liver cirrhosis secondary to alcoholic hepatitis. Her medical records included grade I-II esophageal varices, atrial and right ventricular dilation, and mixed valvulopathies including moderate mitral, tricuspid, and pulmonary regurgitations. The patient’s preoperative model for end-stage liver disease (MELD) score was 16 points.

**Figure 2:**
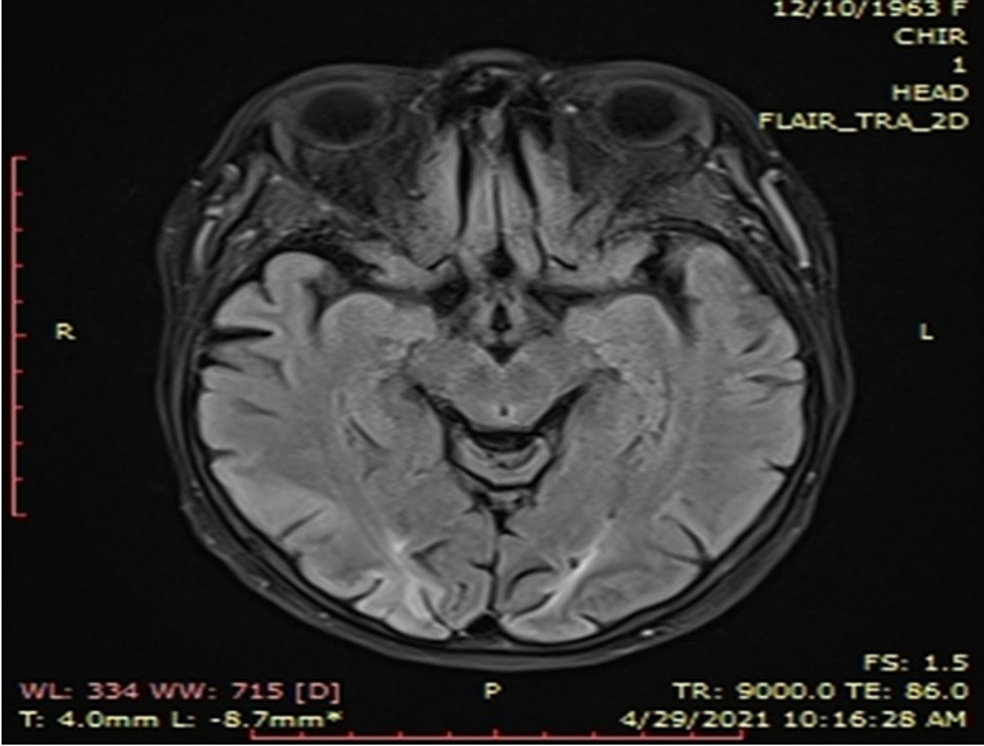
Head MRI scan showing bilateral parieto-occipito-frontal subacute cortical ischemia

The patient received 20 mg basiliximab and 500 mg methylprednisolone intraoperatively. Immunosuppression was maintained with tacrolimus and MMF, with dosing guided by the blood count analysis and liver function tests. A second dose of basiliximab was administered on the fourth postoperative day. The patient had a favorable postoperative outcome, and she was discharged from the ICU facility five days later. One week after liver transplantation, the patient showed transitory neurologic impairment consisting of left transitory hemiplegia. The patient was readmitted to the ICU. The MRI-scan identified bilateral parieto-occipito-frontal subacute cortical ischemia (Figure 2).

**Figure 3:**
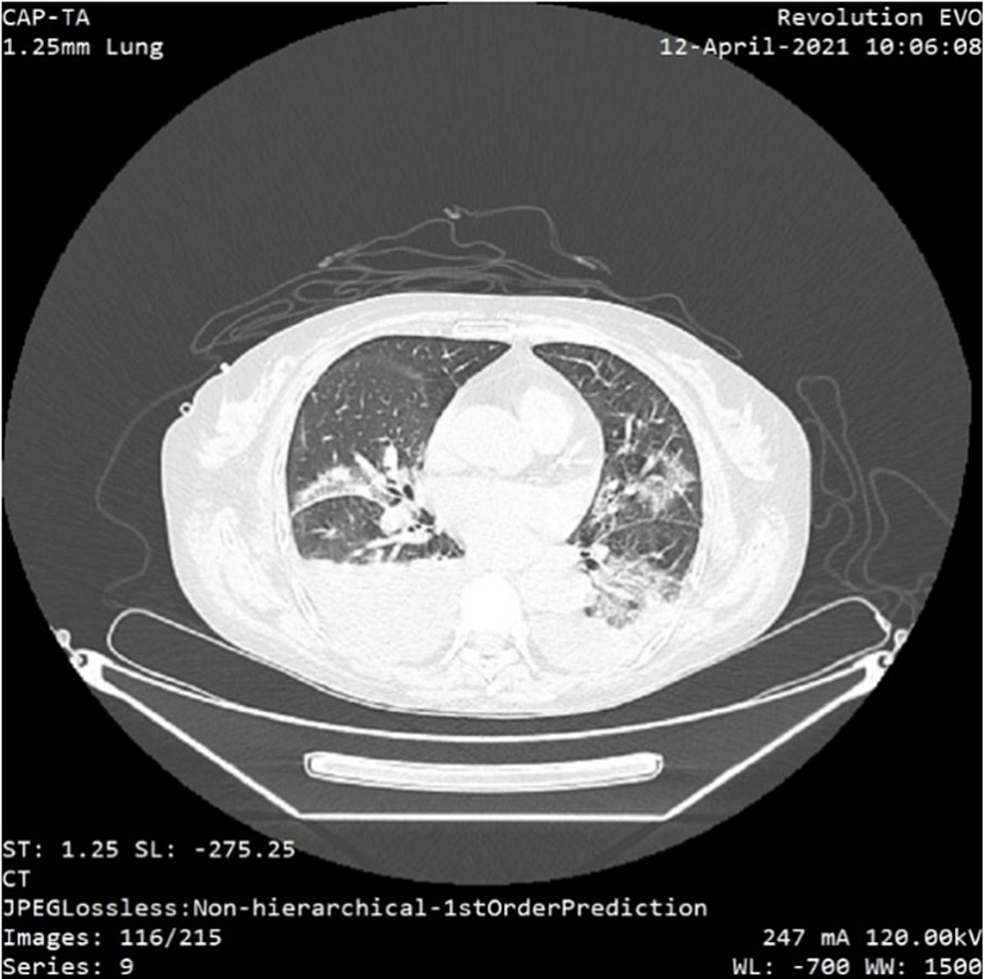
Thorax CT scan revealing moderate bilateral pleural effusions and typical glass opacities, peripheral distributed in approximately 50% of the lung parenchyma

Shortly after readmission, the patient developed acute hypoxemic respiratory failure. She tested positive for COVID-19. The native-phase lung CT scan identified a severe form of COVID-19 pneumonia, with 50% lung involvement (Figure 3). The patient received oxygen via a non-rebreather face mask with a maximum flow of 8 l/min, displaying good tolerance.

Moreover, specific antiviral therapy was initiated. The patient was given remdesivir and corticosteroids for 10 days, in parallel with specific immunosuppression - MMF and tacrolimus. The complete resolution of the motor and sensory deficits was observed two days after the ICU readmission. She was discharged from the ICU seven days after the positive COVID-19 test. After another 26 days, she was discharged from the hospital, with no other respiratory or neurologic features. 

Case 3

A 50-year-old female patient underwent orthotopic liver transplantation for Wilson’s disease. Her medical records revealed asthma and non-insulin-dependent type II diabetes mellitus. The preoperative MELD score was 32 points. She received standard intraoperative immunosuppression comprising 20 mg basiliximab and 500 mg methylprednisolone. For the maintenance of immunosuppression, a second dose of basiliximab was administered on the fourth postoperative day, and a combination of MMS and tacrolimus was initiated immediately after surgery, with dosing guided by the blood count analysis and liver function tests. After a favorable postoperative evolution, she was discharged, with oral tacrolimus prescribed as primary liver graft rejection prophylaxis.

**Figure 4:**
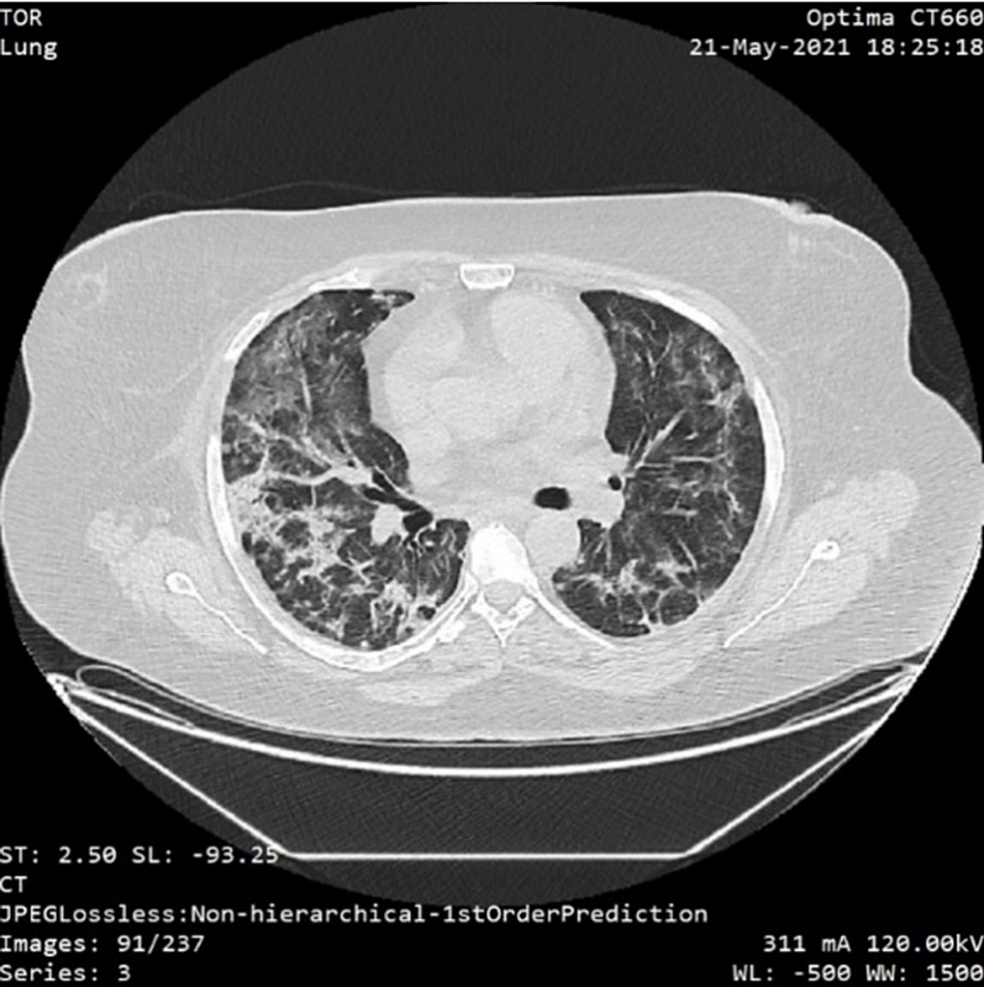
Thorax CT scan showing typical fibrotic lesions associated with bronchiectasis extensive distributed to the right lung and 2/3 of the left lung (over 75% lung involvement)

Eighteen years later, the patient was diagnosed with COVID-19, in the context of the SARS-CoV-2 pandemic. Due to acute hypoxemic respiratory failure, the patient was admitted to the ICU. No other organ dysfunction was discovered. The lung CT scan revealed major lung involvement (over 75%) (Figure 4).

Specific antiviral therapy with remdesivir and corticosteroids was initiated. Her symptoms subsided after seven days of ICU stay, of which five days with continuous positive airway pressure (CPAP) therapy and two days with supplemental oxygen via a non-rebreather face mask. She was discharged from the hospital after a total stay of 14 days, with no other respiratory features.

## Discussion

The functional impact of COVID-19 pneumonia in the patients presented above was minor, regardless of the time since the transplantation. Two patients benefited from supplemental oxygen via a facial non-rebreather mask and one patient required five days of non-invasive mechanical ventilation. Two of them needed management in the ICU. No patient needed invasive mechanical ventilation and none returned with any features of the post-COVID-19 syndrome. Thus, we described the impact of COVID-19 pneumonia on acquired immunosuppression liver-transplanted patients at relevant increasing time points (i.e., days, months, or even years) after surgery, considering variations in immunosuppression intensity after the surgical event, a key issue. 

At the time of admission, our patients displayed the following symptoms: fever, cough, dyspnea and tachypnea, pale skin, and mucous membranes. Moreover, we identified increased levels of c-reactive protein (CRP), D-dimers, ferritin, and typical blood count changes. Only one patient had shown reversible neurologic impairment, with subacute ischemic changes on MRI scan.

Our results are in accordance with those published by Kulkarni et al. [12]. They have shown that the most common symptoms in liver transplanted patients were fever (49.7%), cough (43%), and dyspnea (30%). They also identified a specific increase of some biochemical markers, such as ferritin, D-Dimers, and CRP [12]. Thus, our descriptive case series raises clinician awareness regarding such symptoms in liver transplanted patients. 

Moreover, a meta-analysis revealed that the outcomes of patients infected with SARS-CoV-2 a few days or weeks after liver transplantation was similar to other categories of patients, with no impact on mortality [13]. Our case series is, in part, in accordance with those findings. Conversely, the patient in case 3 developed COVID-19 years after transplantation, whose clinical circumstance is, however, not included in the above-mentioned meta-analysis results.

Some clinical studies suggested changes in immunosuppression regimen for liver-transplanted patients with COVID-19 pneumonia [14-16]. Cyclosporine and tacrolimus are strong calcineurin inhibitors and are considered fundamental immunosuppressant drugs in liver transplant patients [7]. Tacrolimus binds to immunophilin receptors (FK 506-binding proteins {FKBPs}), blocks calcineurin phosphatase activity, and thus inhibits the transcription of genes involved in IL-2 synthesis [17]. Its effect on COVID-19 is still unknown; however, some data suggest that calcineurin inhibitors have a direct antiviral effect [18]. All our patients were on tacrolimus therapy when admitted for COVID-19 pneumonia. This could explain the discrepancy between the clinical presentation and structural lung changes observed in our patients.

MMF is an antibiotic isolated from Penicillium species that also has immunosuppressive properties [19]. The drug acts by selectively inhibiting purine synthesis, therefore being a potent inhibitor of B and T lymphocyte proliferation [19]. MMF plays an important role in the treatment of acute rejection [20]. Combining tacrolimus and MMF results in a safe reduction in the required doses of CNI [20].

CNI and MMF doses were either reduced or discontinued in most of the patients with COVID-19 pneumonia, even though, due to its inhibitory effect on T-cell activation phase, tacrolimus has been associated with an improved survival rate [15,16,21]. Although immunosuppression may prolong viral shedding in liver transplanted recipients with COVID-19, complete discontinuation of immunosuppression should be discouraged [22]. In accordance with those findings, our patients received the usual dosage regimen for tacrolimus as primary prophylaxis against host vs. graft reaction, whereas MMF was discontinued on approximately 70% of the days of hospitalization in the patient of case 1, while the patient of case 2 did not receive it on approximately 51% of the days.

Patient in case 3 received tacrolimus in monotherapy as prophylaxis against host vs. graft reaction in accordance with local protocol. The liver-transplanted patients receive MMF either in perioperative settings or in case of acute rejection. The patients were discharged between the 14th and 26th day after COVID-19 confirmation. Due to the heterogeneity of the data in the literature regarding median hospital stay for immunosuppressed patients, no conclusion could be drawn regarding the severity of the illness based on this criterion. Moreover, prolonged viral shedding in our patients could not be excluded.

Basiliximab is a murine/human chimeric monoclonal antibody that binds to the alfa chain of interleukin-2 receptors (CD25 antigen), expressed on the surface of T-lymphocytes in response to antigenic aggression [23]. Basiliximab specifically targets the high-affinity CD25 antigen on activated T-cells, thus preventing interleukin-2 binding, a critical signal for T-cell proliferation in the cellular immune response involved in perioperative allograft rejection [23]. Comparing the outcomes of patients in cases 1 and 2, who recently received basiliximab, with the patient in case 3, who did not receive basiliximab, we could mention that immunosuppression with basiliximab did not impact the outcome of our patients. Further clinical studies are needed in order to confirm our observation. 

Besides various approaches for primary prophylaxis against graft rejection in liver-transplanted patients, specific antiviral therapy against SARS-CoV-2 infection should be added in accordance with the local guidelines [24]. The most used therapy at that time involved remdesivir and systemic corticosteroids. Remdesivir is a prodrug which, after undergoing intracellular metabolic conversion to a triphosphate nucleoside analog, competes with endogenous adenosine triphosphate for the integration into nascent viral RNA strands by way of RNA-dependent RNA polymerase; this analog proved to be more effectively incorporated by the polymerase, compared to the natural nucleotides [25].

Remdesivir has a short plasma half-life (approx. one hour), however, its analog metabolites have significantly longer half-lives, of up to 35 hours, which enables the administration of remdesivir as a single daily dose - 200 mg loading on the first day of the regimen, followed by 100 mg daily from day two up to five or 10, depending on the patient’s responsiveness to therapy [25]. One of the most relevant side effects in this context is represented by the abnormal liver function parameters [25]. Our study identified severe structural lung involvement in all patients, despite using systemic corticosteroids and remdesivir, which evolved, however, towards a good outcome. Some of the most important co-morbidities associated with the worse outcomes of COVID-19 include obesity, the presence of malignancy, and end-stage kidney disease [25-27].

Regarding the neurological impairment observed in the patient of case 2, it could be attributed to COVID-19 infection, as the result of multiple possible mechanisms, including "cytokine storm", virus-induced hypercoagulability, or direct infection of the central nervous system [28]. Viral neuroinvasion can be achieved in several ways, including transsynaptic transfer between infected neurons, olfactory nerve or vascular endothelial infection, or leukocyte migration across the blood-brain barrier [28]. Several neuropathologic studies have revealed different patterns of central nervous system (CNS) damage, especially ischemic and hemorrhagic, but also inflammatory lesions [28]. Our patient exhibited minor ischemic diffuse lesions.

## Conclusions

Although immunosuppressant therapy was inconsistent during hospitalization, our patients had a favorable outcome, requiring no invasive mechanical ventilation. They were quickly diagnosed and treated from the onset of symptoms. While the mortality in COVID-19 immunosuppressed patients is not higher than in the general population, clinicians should be made aware of the potentially severe lung complications associated with this complex pathology.
